# Supplementary material for: Oxytocin signaling in the medial amygdala is required for sex discrimination of social cues
Source: eLife. 2017 Dec 12;6:e31373. doi: 10.7554/eLife.31373 (PMC5768418; doi:10.7554/eLife.31373)
Supplement: Transparent reporting form [file elife-31373-transrepform.docx]

***eLife’s* transparent reporting form**

We encourage authors to provide detailed information *within their submission* to facilitate the interpretation and replication of experiments. Authors can upload supporting documentation to indicate the use of appropriate reporting guidelines for health-related research (see [EQUATOR Network](http://www.equator-network.org/%20)), life science research (see the [BioSharing Information Resource](https://biosharing.org/)), or the [ARRIVE guidelines](http://www.plosbiology.org/article/info:doi/10.1371/journal.pbio.1000412) for reporting work involving animal research. Where applicable, authors should refer to any relevant reporting standards documents in this form.

If you have any questions, please consult our Journal Policies and/or contact us: [editorial@elifesciences.org](mailto:editorial@elifesciences.org).

**Sample-size estimation**

- You should state whether an appropriate sample size was computed when the study was being designed
- You should state the statistical method of sample size computation and any required assumptions
- If no explicit power analysis was used, you should describe how you decided what sample (replicate) size (number) to use

Please outline where this information can be found within the submission (e.g., sections or figure legends), or explain why this information doesn’t apply to your submission:

Sample size was determined by comparing to those used in similar experiments manipulating neuronal activity using chemogenetic methods, monitoring changes in mouse social behavior using behavioral paradigms, and recording neuronal activities using electrophysiology respectively. In all cases, viral injections were simultaneously done in for the entire cohort, and to maximize animal use the number of animals also depended on the litter size.

**Replicates**

- You should report how often each experiment was performed
- You should include a definition of biological versus technical replication
- The data obtained should be provided and sufficient information should be provided to indicate the number of independent biological and/or technical replicates
- If you encountered any outliers, you should describe how these were handled
- Criteria for exclusion/inclusion of data should be clearly stated
- High-throughput sequence data should be uploaded before submission, with a private link for reviewers provided (these are available from both GEO and ArrayExpress)

Please outline where this information can be found within the submission (e.g., sections or figure legends), or explain why this information doesn’t apply to your submission:

Numbers of independent biological replicates used in the behavioral tests are listed in the figures and the text.

Numbers of mice used in the mapping of OXT outputs from the PVN and the SON are listed in the figure legend.

No outliers were encountered.

The numbers of biological replicates for electrophysiological experiments are listed in the methods and the figure legends. As is standard for electrophysiological experiments, the number of units recorded from each animal varied some. Only single units were included in the final data analysis; however, multi-unit responses were analyzed in the same manner and produced nearly identical results on all major points of discussion.

**Statistical reporting**

- Statistical analysis methods should be described and justified
- Raw data should be presented in figures whenever informative to do so (typically when N per group is less than 10)
- For each experiment, you should identify the statistical tests used, exact values of N, definitions of center, methods of multiple test correction, and dispersion and precision measures (e.g., mean, median, SD, SEM, confidence intervals; and, for the major substantive results, a measure of effect size (e.g., Pearson's r, Cohen's d)
- Report exact p-values wherever possible alongside the summary statistics and 95% confidence intervals. These should be reported for all key questions and not only when the p-value is less than 0.05.

Please outline where this information can be found within the submission (e.g., sections or figure legends), or explain why this information doesn’t apply to your submission:

Statistical methods including methods of multiple test correction are described in the methods section and on a case-by-case basis in the results section. Multiple test corrections were used in all cases for which multiple comparisons were made.

The specific statistical test used, exact values of N, exact p-values and standard deviation in a sample set of data (STDEV) are consistently listed in the results text describing each experiment.

(For large datasets, or papers with a very large number of statistical tests, you may upload a single table file with tests, Ns, etc., with reference to sections in the manuscript.)

**Group allocation**

- Indicate how samples were allocated into experimental groups (in the case of clinical studies, please specify allocation to treatment method); if randomization was used, please also state if restricted randomization was applied
- Indicate if masking was used during group allocation, data collection and/or data analysis

Please outline where this information can be found within the submission (e.g., sections or figure legends), or explain why this information doesn’t apply to your submission:

In behavioral tests, groups are based on different genotypes, different types of virus infection, and different drug treatment. Mice of different genotypes, but the same genetic background, were used for experiments. When subjecting mice of the same genotype to different virus injection or drug treatment, mice were randomly distributed into different treatment groups, and received viral injection/drug treatment at the same age.

**Additional data files (“source data”)**

- We encourage you to upload relevant additional data files, such as numerical data that are represented as a graph in a figure, or as a summary table
- Where provided, these should be in the most useful format, and they can be uploaded as “Source data” files linked to a main figure or table
- Include model definition files including the full list of parameters used
- Include code used for data analysis (e.g., R, MatLab)
- Avoid stating that data files are “available upon request”

Please indicate the figures or tables for which source data files have been provided:

The custom matlab scripts used for analysis of electrophysiological data can be downloaded from the following site: <https://github.com/joebergan/Spike-sorting>

All source data will be available upon request.
